# Agreement in Biometric Parameters Between Swept-Source–Optical Coherence Tomography and Optical Low-Coherence Interferometry: Insights into Clinical Precision

**DOI:** 10.3390/jcm14051407

**Published:** 2025-02-20

**Authors:** Mihnea Munteanu, Leila Al Barri, Simona Stanca, Valeria Mocanu, Cosmin Rosca, Nicolae-Constantin Balica, Horia T. Stanca

**Affiliations:** 1 Ophthalmology Department, “Victor Babes” University of Medicine and Pharmacy, 300041 Timisoara, Romania; mihnea.munteanu@umft.ro (M.M.); mocanu.valeria@umft.ro (V.M.); cosmin.rosca@umft.ro (C.R.); 2 Oftalmo Sensory-Tumor Research Center—ORL (EYE-ENT), “Victor Babes” University of Medicine and Pharmacy, 300041 Timisoara, Romania; 3 Pediatrics Department, “Carol Davila” University of Medicine and Pharmacy, 020022 Bucharest, Romania; simona.stanca@umfcd.ro; 4 ENT Department, “Victor Babes” University of Medicine and Pharmacy, 300041 Timisoara, Romania; balica@umft.ro; 5 Ophthalmology Department, “Carol Davila” University of Medicine and Pharmacy, 050474 Bucharest, Romania; horia.stanca@umfcd.ro

**Keywords:** swept-optical coherence tomography, optical low-coherence interferometry, biometric measurements, axial length, keratometry, astigmatism, anterior chamber depth, clinical application

## Abstract

**Background/Objectives:** Accurate biometric measurements are critical for achieving optimal refractive outcomes in cataract surgery. This study evaluated the agreement of biometric measurements between a swept-source optical coherence tomography (SS–OCT) biometer (Argos^®^, Movu Inc.) and an optical low-coherence interferometry (OLCI) biometer (Aladdin^®^, Topcon Corp.). Parameters analyzed included axial length (AL), anterior chamber depth (ACD), lens thickness (LT), keratometry (K1, K2), and white-to-white corneal diameter (WTW). **Methods:** A total of 170 eyes were examined, and agreement was assessed using Bland–Altman analysis, intraclass correlation coefficients (ICCs), and Pearson correlation coefficients. **Results:** Excellent agreement was observed for AL (ICC = 0.975), ACD (ICC = 0.960), LT (ICC = 0.951), K1 (ICC = 0.921), and K2 (ICC = 0.927). Moderate agreement was found for astigmatism axis (ICC = 0.655) and cylinder power (ICC = 0.891). Poor agreement was noted for astigmatism-related Jackson cross-cylinder vectors J0 (ICC = 0.334) and J45 (ICC = −0.311), as well as for WTW (ICC = 0.338). Bland–Altman plots demonstrated narrow limits of agreement for most parameters, with mean differences of 0.009 mm for AL and 0.06 mm for ACD. **Conclusions:** Both devices demonstrated high degrees of agreement for core biometric parameters, supporting their clinical interchangeability. However, the variability in WTW and astigmatism-related measurements highlights the need for caution when precise corrections are required.

## 1. Introduction

Accurate ocular biometry is indispensable in modern ophthalmology, particularly for intraocular lens (IOL) power calculation during cataract surgery and refractive procedures. This precision highlights the clinical goal of minimizing refractive errors, thereby enhancing both surgical outcomes and patient satisfaction [1,2]. Over recent decades, advances in optical biometry have largely replaced earlier ultrasound techniques, offering non-contact methods with improved accuracy and reproducibility [2,3].

Technological innovations have introduced various measurement systems, including partial-coherence interferometry (PCI), optical low-coherence reflectometry (OLCR), optical low-coherence interferometry (OLCI) and swept-source optical coherence tomography (SS–OCT). While PCI, pioneered by the IOLMaster series, set a gold standard for decades, limitations in dense cataracts prompted the development of OLCR/OLCI and SS–OCT technologies, which achieve higher penetration and resolution [4,5]. These advancements are critical, as accurate axial length (AL) and keratometry measurements contribute to up to 54% of refractive prediction errors in cataract surgery [2,6,7].

SS–OCT has emerged as a transformative innovation, leveraging longer wavelengths for superior tissue penetration and imaging depth. Devices like the IOLMaster (Carl Zeiss Meditec AG, Jena, Germany) or Argos (Movu, Inc., Fort Worth, TX, USA) not only ensure high reproducibility but also allow the visualization of entire ocular structures, offering diagnostic advantages beyond biometric measurements [8,9]. Comparative studies have consistently shown the reliability of SS–OCT across diverse patient populations, including those with challenging conditions such as dense cataracts [10,11].

Despite the consensus on the superior imaging capabilities of SS–OCT, the agreement between this technology and OLCI remains a critical area of investigation. OLCI devices, such as the Lenstar (Haag-Streit AG, Köniz, Switzerland) or Aladdin (Topcon Corporation, Tokyo, Japan), have long demonstrated high repeatability and ease of use, particularly in cases with clear optical pathways [12,13]. However, head-to-head comparisons reveal nuanced differences in measurements such as anterior chamber depth (ACD) and lens thickness (LT), which may influence IOL power predictions [14,15].

The potential impact of these measurement differences extends to clinical outcomes. Studies exploring IOL power calculations based on formulas like Barrett Universal II and Haigis underscore the importance of precise biometry [16,17]. While both OLCR/OLCI and SS–OCT technologies achieve clinically acceptable levels of agreement, subtle variations in parameters such as AL and ACD may affect refractive outcomes in specific patient cohorts [18,19]. This study extends beyond a technical comparison of SS–OCT and OLCI devices by evaluating their clinical impact on IOL power calculations, astigmatism assessment, and biometric accuracy in different patient subgroups. Given the significance of precise biometry in surgical outcomes, the findings provide critical guidance for optimizing device selection in clinical practice.

This study aims to compare the agreement between a SS–OCT and an OLCI biometer, focusing on their clinical utility in precise ocular biometric measurements critical for cataract surgery and refractive outcomes.

## 2. Materials and Methods

### 2.1. Study Design, Ethics, and Informed Consent

This retrospective, observational study analyzed refractive and visual outcomes in patients who had undergone successful cataract surgery. The research was conducted within the Ophthalmology Department at the “Victor Babeș” University of Medicine and Pharmacy in Timișoara, Romania, between January 2022 and June 2024. The study complied with the ethical standards set forth by the Declaration of Helsinki. Given its retrospective nature, the institutional ethics committee waived the need for specific approval. Additionally, all participants had provided informed consent for their surgeries, which included permission for the use of their data in research. Throughout the study, patient confidentiality was rigorously protected.

### 2.2. Subjects and Inclusion/Exclusion Criteria

Patients selected for this study were those who had undergone uncomplicated cataract surgery with IOL implantation. All biometric measurements analyzed in this study were obtained preoperatively, before intraocular lens (IOL) implantation. No post-operative measurements were included in the analysis. Biometric data collection using either SS–OCT (Movu, Inc., Fort Worth, TX, USA) or OLCI (Aladdin, Topcon Corporation, Tokyo, Japan) devices was guided by clinical workflow and equipment availability, ensuring a comparable distribution of cases for comparison.

Eligibility criteria included the following: (1) eyes with no history of trauma or injury; (2) no previous ocular surgeries; (3) absence of systemic conditions that could affect ocular health; (4) preoperative corrected distance visual acuity (CDVA) of 0.4 logMAR or better; (5) corneal astigmatism with a regular pattern. The exclusion criteria were as follows: (1) a history of corneal ectasia; (2) significant dry-eye syndrome; (3) retinal disorders; (4) previous refractive surgery.

### 2.3. Procedure

All patients underwent a detailed preoperative ophthalmic examination including biometric measurements with both SS–OCT and OLCI devices. The measurements included AL, ACD, LT, keratometry (flat, steep, and axis), and white-to-white corneal diameter (WTW). These variables were measured sequentially using both devices, with the order of device usage randomized to minimize bias. To ensure consistent tear-film quality, patients were instructed to blink before each measurement. For each patient, three consecutive scans were performed and the average values of the results were analyzed.

### 2.4. Biometers

Biometric measurements were conducted using two advanced optical devices. The Argos^®^ biometer (Movu Inc., Komaki, Japan), which operates on SS–OCT technology, utilizes a 1060 nm light source and incorporates segmental refractive indices (cornea: 1.376; aqueous and vitreous: 1.336; lens: 1.410) to enhance measurement accuracy. On the other hand, the Aladdin^®^ biometer (Topcon Corp., Tokyo, Japan) employs OLCI with an 830 nm light source and a Placido disk for keratometry. It is important to note that the segmental refractive index settings in the SS–OCT device are predefined, whereas the OLCI device does not allow manual adjustments. Variations in assumptions related to the refractive index may have contributed to the minor differences observed in measurements of axial length and anterior chamber depth, particularly in cases with altered lenses or corneal conditions.

### 2.5. Agreement Analysis and Statistical Methods

The agreement between biometric measurements obtained from the SS–OCT and OLCI devices was evaluated for key parameters, including flat and steep keratometry, AL, ACD, LT, and WTW. Bland–Altman plots were used to assess the differences between the devices, providing a visualization of the mean difference and 95% limits of agreement (LoA). Astigmatism parameters were analyzed using double-angle plots (described by Abulafia et al. [20]), which mapped astigmatism power and axis with Jackson cross-cylinder vectors (J0, J45). The yellow data points represent individual astigmatic measurements, while the centroid and 95% confidence ellipses of the dataset were plotted for each biometer to evaluate the distribution and agreement of astigmatism-related measurements.

Statistical analyses were performed using SPSS version 29 (IBM Corp., Armonk, NY, USA). Descriptive statistics, including means, standard deviations, and ranges, were calculated for all variables. The normality of the data was assessed using the Kolmogorov–Smirnov test. Paired *t*-tests were employed to detect significant differences between the two devices. Pearson correlation coefficients were computed to quantify linear relationships, and intraclass correlation coefficients (ICCs) were used to evaluate reliability and agreement between the two measurement methods. ICC values were categorized as poor (<0.5), moderate (0.5–0.75), good (0.75–0.9), or excellent (>0.9).

## 3. Results

The study evaluated a total of 170 eyes from 102 patients, with a mean age of 68.88 ± 10.82 years, ranging from 40 to 86 years. The cohort comprised 57 males (55.9%) and 45 females (44.1%). The preoperative refractive data showed a mean spherical equivalent of 0.36 ± 3.17 D, with individual values ranging from −13.13 to 8.63 D. The mean sphere was 0.46 ± 3.07 D, with a range from −12.00 to 8.00 D, while the mean cylinder was −0.10 ± 1.57 D, ranging from −4.50 to 4.25 D. The axis of astigmatism varied widely, with a mean of 80.59° ± 60.45° (range: 0° to 180°). Visual acuity, assessed using CDVA (LogMAR), had a mean value of −0.39 ± 0.31, with values between 0.00 and 1.70. Intraocular pressure ranged from 10.0 to 27.5 mmHg, with a mean of 15.97 ± 3.44 mmHg. These descriptive statistics provide a comprehensive overview of the baseline characteristics of the study population and their refractive status and serve as the foundation for the comparative analysis between SS–OCT and OLCI measurements.

Table 1 presents a detailed comparison of biometric measurements between the SS–OCT and OLCI. The results demonstrate high consistency between the two modalities, with significant differences observed in certain variables.

Table 2 summarizes the analysis of agreement between the SS–OCT and OLCI biometers. The mean differences for most variables were minimal, indicating close agreement. The results indicate a strong agreement between SS–OCT and OLCI for most variables, particularly in axial and keratometry measurements, but highlight certain discrepancies in astigmatism-related parameters and WTW measurements.

**Figure 1:**
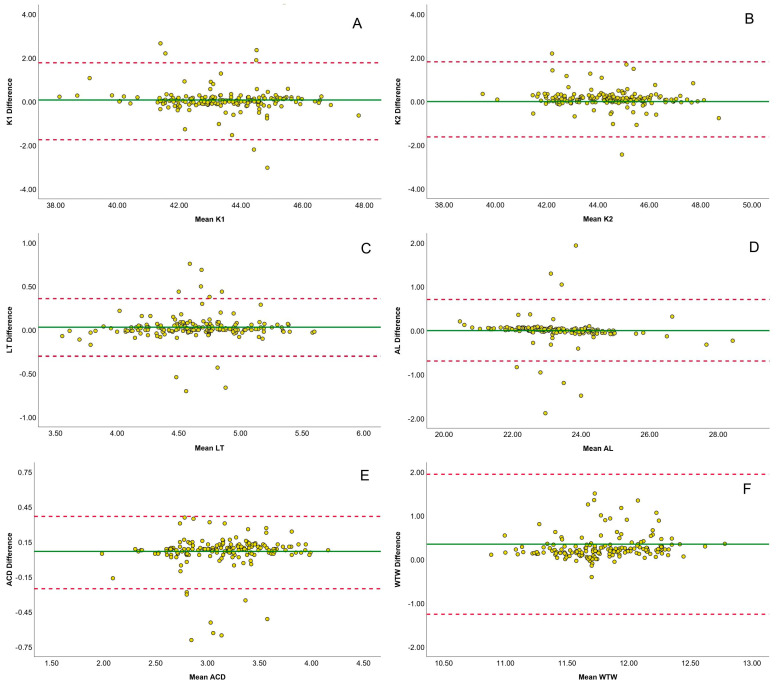
Bland–Altman plots comparing biometric measurements between SS–OCT and OLCI. Each yellow circle represent individual difference measurement of each comparison. The plots depict the agreement between SS–OCT and OLCI for flat keratometry (K1, Panel (**A**)), steep keratometry (K2, Panel (**B**)), lens thickness (LT, Panel (**C**)), axial length (AL, Panel (**D**)), anterior chamber depth (ACD, Panel (**E**)), and white-to-white distance (WTW, Panel (**F**)). The solid green line represents the mean difference, and the dashed red lines indicate the limits of agreement (LoA).

Figure 1 contains Bland–Altman plots comparing the agreement between SS–OCT and OLCI biometers for various parameters. Each subplot represents the mean differences between the devices plotted against their averages, with the solid green line indicating the mean difference and the dashed red lines representing the limits of agreement (LoA). Flat keratometry yielded minimal mean differences, with most data points lying within the LoA (−1.74 to 1.78 mm), indicating strong agreement between devices. Steep keratometry data exhibit a similar pattern, with narrow LoA (−1.62 to 1.82 mm) and data closely clustering around the mean difference. LT data show excellent agreement, with a mean difference near zero and narrow LoA (−0.30 to 0.36 mm), confirming their high consistency. AL data show tight clustering around the mean difference, with most values within the LoA (−0.69 to 0.71 mm), indicating robust agreement. ACD data have a narrow LoA (−0.25 to 0.37 mm) and a minimal mean difference, indicating strong reliability. WTW data show broader variability, with LoA ranging from −1.25 to 1.95 mm, reflecting lower agreement compared to the other parameters.

**Figure 2:**
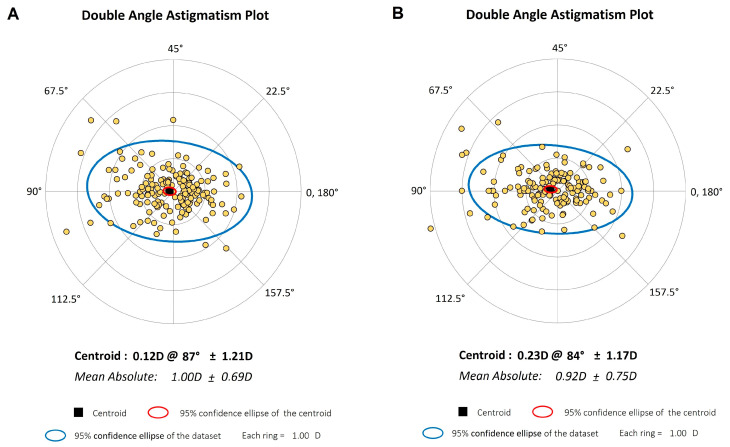
Double-angle astigmatism plots comparing SS–OCT and OLCI. Polar plots show the astigmatic components for SS–OCT (Panel (**A**)) and OLCI (Panel (**B**)). The yellow datapoints represent individual astigmatic measurements; centroid positions are shown in black; 95% confidence ellipses are given for the dataset and the centroid. The plots highlight the distribution of astigmatism magnitude and axis for each device.

Figure 2 presents the double-angle astigmatism plots for comparing astigmatic components between the SS–OCT and OLCI. These polar plots provide a visual representation of the magnitude and axis of astigmatism, with each data point corresponding to an eye’s astigmatic value. The double-angle plots highlight strong agreement between SS–OCT and OLCI data for astigmatic measurements, as both datasets show comparable centroids and mean absolute astigmatism. However, OLCI data exhibit slightly greater variability, as evidenced by the broader 95% confidence ellipse. These findings suggest that while both devices provide reliable astigmatism data, SS–OCT might offer slightly more precision in capturing astigmatic components.

Table 3 provides a detailed analysis of AL measurements across three groups—short eyes (<23.00 mm), medium eyes (≥23.00 and <24.00 mm), and long eyes (≥24.00 mm)—comparing data obtained from SS–OCT and OLCI. The results highlight strong agreement between SS–OCT and OLCI for AL measurements in short and long eyes, as evidenced by high Pearson correlations and ICC values above 0.940. The agreement is particularly robust in long eyes, for which the ICC reaches 0.984. In contrast, medium eyes show lower agreement, with a weak correlation and an ICC of 0.319, possibly due to greater variability in this group.

## 4. Discussion

The results of this study demonstrate strong overall agreement between SS–OCT and OLCI for biometric measurements. Key parameters such as flat and steep keratometry, AL, LT, and ACD exhibited minimal mean differences, high Pearson correlations, and excellent ICC, confirming the reliability of both devices. However, discrepancies were noted in astigmatism-related parameters, including the axis of astigmatism, cylinder power, and Jackson cross-cylinder components, for which agreement was moderate to low. Additionally, WTW showed broader limits of agreement and lower ICC values. Subgroup analysis of AL revealed robust agreement for short and long eyes, with reduced consistency in medium eyes. These findings highlight the comparability of SS–OCT and OLCI in most biometric measurements, while also identifying specific areas where differences may influence clinical decision-making.

While the statistical agreement between SS–OCT and OLCI was high for key parameters, clinical relevance must be assessed individually. Small differences in axial length and anterior chamber depth, though statistically nonsignificant, may affect intraocular lens power calculations, particularly in patients who have undergone refractive surgery or in those with extreme biometrics. Similarly, the variability in WTW and astigmatism parameters suggests that caution is warranted in applications requiring high precision, such as toric IOL selection.

### 4.1. Axial Length

The current study demonstrated excellent agreement in AL measurements between SS–OCT and OLCI devices, with a mean difference of 0.009 mm (SD ± 0.36) and an ICC of 0.975. These results align with findings from Hoffer et al. [10], who observed negligible differences in AL measurements between the IOLMaster 700 and Lenstar LS 900, with ICCs > 0.99. Similarly, Gao et al. [2] reported strong agreement with Bland–Altman limits of agreement within ±0.05 mm to ±0.07 mm, highlighting the high precision of these technologies. However, subgroup analysis in this study highlighted minor discrepancies in medium and long eyes, which were less frequently discussed in prior literature.

### 4.2. Anterior Chamber Depth

This study observed a small but statistically significant difference in ACD measurements, with SS–OCT consistently measuring deeper values (mean difference 0.06 mm, SD ± 0.16, ICC = 0.960). This finding echoes the results of Kurian et al. [12] and Hoffer et al. [10], who both reported deeper ACD measurements with SS–OCT compared to OLCR, though the differences remained clinically nonsignificant. The ability of SS–OCT to penetrate dense media may partly explain these differences, as noted by Kanclerz et al. [21].

### 4.3. Lens Thickness

Agreement in LT measurements between the two devices was excellent, with a mean difference of 0.03 mm (SD ± 0.17) and ICC of 0.951. This is consistent with findings from Yu et al. [13] and Kunert et al. [5], who also observed high concordance in LT values between SS–OCT and OLCR/OLCI devices. These findings highlight the reliability of both technologies for preoperative evaluations in which precise LT measurements are critical.

### 4.4. Keratometry and Astigmatism

Keratometry values (flat K1 and steep K2) displayed high levels of agreement, with ICC values of 0.921 and 0.927, respectively, and mean differences of 0.02 mm (K1) and 0.10 mm (K2). These results are comparable to those of Gao et al. [2], who demonstrated similar agreement between SS–OCT and OLCR/OLCI devices, with ICCs > 0.98 for all keratometric parameters.

Astigmatism vectors (J0 and J45) exhibited variable agreement, with J0 showing moderate agreement (ICC = 0.334) and J45 displaying lower reliability (ICC = −0.311). This variability may be attributed to differences in the algorithmic processing of astigmatism data between devices, as also noted in studies by Zharei Ghanavati et al. [11] and Passi et al. [14]. Employing double-angle vector representation to analyze differences in astigmatism measurements could provide a more nuanced understanding of the observed variability and improve comparative analysis between devices. SS–OCT uses Fourier-domain scanning, while OLCI relies on Placido-disc topography, which is more sensitive to tear-film irregularities and fixation errors; these may thus influence astigmatism readings. These differences are clinically relevant for toric IOL alignment, in which small variations can impact postoperative refractive outcomes.

### 4.5. White-to-White

WTW measurements showed notable differences, with SS–OCT measuring larger values (mean difference 0.35 mm, SD ± 0.82, ICC = 0.338). El Chebab et al. [6] also reported differences in WTW between these devices but attributed them to variations in measurement algorithms and device design. Yu et al. [13] similarly observed discrepancies in WTW, emphasizing the need for device-specific calibration in clinical practice. For WTW measurements, SS–OCT cross-sectional imaging provides a consistent limbal-border detection, whereas OLCI’s projection-based approach may be affected by illumination and edge-detection variability. Given the role of WTW in phakic IOL sizing, inconsistent values could influence lens selection, increasing the risk of vaulting or decentration. Clinicians should be aware of these inter-device variations, particularly in precision-driven procedures such as toric IOL implantation and phakic IOL selection. Cross-validation of biometric measurements and preoperative aberrometry may help minimize discrepancies, improving surgical accuracy.

Kanclerz et al. [21] and Macalinden et al. [22] emphasized the superior penetration and lower dropout rates of SS–OCT devices compared to OLCR/OLCI, particularly in dense cataracts. These advantages, combined with the robust agreement in key parameters such as AL, ACD, and keratometry, make SS–OCT a versatile choice for clinical settings. However, the discrepancies observed in WTW and astigmatism vectors highlight areas in which further standardization and cross-device calibration may be necessary.

### 4.6. Strengths and Limitations

This study extends prior research [1] by incorporating an expanded astigmatism analysis using Jackson cross-cylinder vectors, performing subgroup analyses based on axial length, and discussing the clinical implications of inter-device variability on IOL power calculations. These distinctions provide a more comprehensive assessment of the interchangeability of SS–OCT and OLCI in biometric measurements for planning of cataract surgeries.

This study has several limitations that should be considered when interpreting the results. First, the sample size, while sufficient for detecting differences in most biometric parameters, may limit the generalizability of findings, particularly for subgroup analyses of medium axial lengths. Second, the study focused exclusively on a single population demographic and thus may not fully capture variability in biometric measurements across different ethnicities, ages, or refractive statuses. Third, potential inter-operator variability in measurement acquisition was not assessed, although this factor could influence the agreement between devices. Additionally, while SS–OCT and OLCI were compared directly, the study did not evaluate their performance against a gold-standard reference method. Moreover, the study did not analyze the potential effect of device-specific artifacts or noise on the measurements, which could play a role in the observed discrepancies.

### 4.7. Future Lines of Research

Future research should explore the integration of SS–OCT and OLCI data through advanced computational methods, such as artificial intelligence or machine learning algorithms, to enhance the precision of biometric measurements. Longitudinal studies could assess how these devices perform over time in tracking progressive conditions, such as keratoconus or cataract development, to determine their reliability in dynamic clinical scenarios. Expanding the scope to include pediatric populations and highly ametropic eyes would provide valuable insights into the versatility of these technologies across a broader range of clinical settings. Lastly, further studies could focus on optimizing device-calibration protocols to minimize variability and improve cross-device standardization in clinical practice.

### 4.8. Clinical Application

The results of this study underline the practical utility of both SS–OCT and OLCI in clinical settings, particularly in preoperative planning for refractive and cataract surgeries. The high degree of agreement observed in key parameters, such as AL, keratometry, and LT, supports the interchangeable use of these devices in routine biometric assessments, as they both yield reliable data for IOL power calculations. Their robust performance in short and long eyes highlights their suitability for a wide range of refractive conditions, while the strong precision in ACD measurements underscores their relevance in surgical planning for anterior segment procedures.

However, the moderate agreement in astigmatism-related parameters suggests that clinicians should carefully evaluate astigmatic data, particularly the axis and cylinder power, when using these devices interchangeably. Additionally, the variability observed in WTW measurements suggests a need for caution in applications requiring precise corneal sizing, such as phakic IOL implantation. The observed differences in biometric measurements may partially stem from device-specific algorithms and measurement protocols, including variations in segmentation techniques, default alignment settings, and refractive-index compensation. These factors should be considered when interpreting inter-device agreement, particularly in parameters for which variability was more pronounced, such as astigmatism and white-to-white corneal diameter.

## 5. Conclusions

This study evaluated the agreement between SS–OCT and OLCI biometric measurements, demonstrating strong concordance in key parameters such as AL, ACD, LT and thus supporting their interchangeability in routine cataract surgery planning. However, moderate discrepancies were observed in astigmatism parameters (J0, J45) and WTW measurements, which may influence toric IOL calculations and phakic IOL sizing.

Clinically, these findings emphasize the importance of cross-validation when selecting biometric devices for precision-driven procedures. The observed differences, though statistically small, may have meaningful refractive implications, particularly in patients who have undergone refractive surgery and cases requiring highly customized IOL power calculations. Surgeons should consider preoperative aberrometry, repeat measurements, or device-specific adjustments to mitigate potential inaccuracies.

## Figures and Tables

**Table 1:** Comparison of biometric measurements between SS–OCT and OLCI biometers.

Variable [Units]	SS–OCT (Mean ± SD Min–Max)	OLCI (Mean ± SD Min–Max)	*p*-Value
K1 [mm]	43.45 ± 1.64 38.24–47.67	43.42 ± 1.70 38.01–48.13	0.335
K2 [mm]	44.45 ± 1.67 39.68–48.61	44.34 ± 1.71 39.33–49.23	0.068
Axis [°]	92.69 ± 55.69 0.00–179.00	83.57 ± 49.16 0.00–178.00	0.014
Cil [D]	−0.99 ± 0.70 −4.25–0.02	−0.92 ± 0.75 −4.14–0.77	0.020
J0 [D]	0.04 ± 0.39 −0.94–1.51	−0.04 ± 0.42 −1.67–1.39	0.021
J45 [D]	−0.07 ± 0.46 −1.55–1.65	−0.02 ± 0.42 −1.25–1.94	0.167
LT [mm]	4.64 ± 0.41 3.52–5.85	4.61 ± 0.39 3.59–5.61	0.009
AL [mm]	23.33 ± 1.15 20.57–28.31	23.32 ± 1.18 20.36–28.54	0.363
ACD [mm]	3.19 ± 0.42 2.01–4.20	3.12 ± 0.40 1.96–4.12	<0.001
WTW [mm]	11.95 ± 0.42 10.94–14.12	11.60 ± 0.83 10.96–12.60	<0.001

SS–OCT: swept-source–optical coherence tomography, SD: standard deviation, OLCI: optical low-coherence interferometry, K1: flat keratometry; K2: steep keratometry; Axis: axis of astigmatism; Cil: cylinder power; J0: 0-degree Jackson cross-cylinder vector; J45: 45-degree Jackson cross-cylinder vector; LT: lens thickness; AL: axial length; ACD: anterior chamber depth; WTW: white-to-white distance.

**Table 2:** Agreement analysis of biometric measurements between SS–OCT and OLCI using Bland–Altman metrics.

Variable [Units]	Mean Difference ± SD	Pearson Correlation (*p*-Value)	ICC (95% CI)	LoA Lower, Upper
K1 [mm]	0.02 ± 0.90	0.855 (<0.001)	0.921 (0.893–0.942)	−1.74, 1.78
K2 [mm]	0.10 ± 0.88	0.865 (<0.001)	0.927 (0.901–0.946)	−1.62, 1.82
Axis [°]	9.11 ± 0.53	0.490 (<0.001)	0.655 (0.532–0.745)	8.07, 10.14
Cil [D]	−0.07 ± 0.45	0.806 (<0.001)	0.891 (0.853–0.920)	−0.95, 0.81
J0 [D]	0.08 ± 0.51	0.201 (0.004)	0.334 (0.098–0.509)	−0.91, 1.07
J45 [D]	−0.04 ± 0.66	−0.135 (0.040)	−0.311 (−0.775–0.032)	−1.33, 1.25
LT [mm]	0.03 ± 0.17	0.907 (<0.001)	0.951 (0.933–0.963)	−0.30, 0.36
AL [mm]	0.009 ± 0.36	0.952 (<0.001)	0.975 (0.966–0.982)	−0.69, 0.71
ACD [mm]	0.06 ± 0.16	0.924 (<0.001)	0.960 (0.946–0.970)	−0.25, 0.37
WTW [mm]	0.35 ± 0.82	0.251 (<0.001)	0.338 (0.102–0.512)	−1.25, 1.95

SS–OCT: swept-Source–optical coherence tomography, SD: standard deviation, OLCI: optical low-coherence interferometry, K1: flat keratometry; K2: steep keratometry; Axis: axis of astigmatism; Cil: cylinder power; J0: 0-degree Jackson cross-cylinder vector; J45: 45-degree Jackson cross-cylinder vector; LT: lens thickness; AL: axial length; ACD: anterior chamber depth; WTW: white-to-white distance.

**Table 3:** Subgroup analysis of axial-length agreement between SS–OCT and OLCI based on eye length.

Axial Length [mm]	SS–OCT Mean ± SD	OLCI Mean ± SD	*p*-Value	Mean Difference ± SD	Pearson Correlation (*p*-Value)	ICC (95% CI)
Short AL [<23.00] (*n* = 68)	22.30 ± 0.58	22.31 ± 0.64	0.379	−0.01 ± 0.29	0.893 (<0.001)	0.941 (0.905–0.964)
Medium AL [≥23.00 and <24.00] (*n* = 59)	23.51 ± 0.36	23.42 ± 0.39	0.093	0.08 ± 0.48	0.190 (<0.074)	0.319 (−0.146–0.595)
Long AL [≥24.00] (*n* = 43)	24.72 ± 0.96	24.78 ± 0.97	0.052	−0.06 ± 0.24	0.969 (<0.001)	0.984 (0.971–0.992)

SS–OCT: swept-source–optical coherence tomography, SD: standard deviation, OLCI: optical low-coherence interferometry AL: axial length.

## Data Availability

The data supporting the findings of this study are available from the corresponding author upon reasonable request.

## Abbreviations

The following abbreviations are used in this manuscript:

ACD	Anterior Chamber Depth
AL	Axial Length
CDVA	Corrected Distance Visual Acuity
D	Diopters
IOL	Intraocular Lens
logMAR	Logarithm of the Minimum Angle of Resolution
K1	Flat Keratometry
K2	Steep Keratometry
LT	Lens Thickness
MAE	Mean Absolute Error
MedAE	Median Absolute Error
ME	Mean Error
OLCI	Optical Low-Coherence Interferometry
OLCR	Optical Low-Coherence Reflectometry
SE	Spherical Equivalent
SS–OCT	Swept-Source Optical Coherence Tomography
UDVA	Uncorrected Distance Visual Acuity
WTW	White-to-White Corneal Diameter
